# MultiBac: from protein complex structures to synthetic viral nanosystems

**DOI:** 10.1186/s12915-017-0447-6

**Published:** 2017-10-30

**Authors:** Martin Pelosse, Hannah Crocker, Barbara Gorda, Paul Lemaire, Jens Rauch, Imre Berger

**Affiliations:** 1 The School of Biochemistry and Bristol Synthetic Biology Centre BrisSynBio, University of Bristol, Tankard’s Close, Bristol, BS8 1TD UK; 2 Geneva Biotech SARL, Avenue de la Roseraie 64, 1205 Genève, Switzerland; 3 Systems Biology Ireland, University College Dublin, Belfield Dublin 4, Republic of Ireland

## Abstract

The MultiBac baculovirus/insect cell expression vector system was conceived as a user-friendly, modular tool-kit for producing multiprotein complexes for structural biology applications. MultiBac has allowed the structure and function of many molecular machines to be elucidated, including previously inaccessible high-value drug targets. More recently, MultiBac developments have shifted to customized baculoviral genomes that are tailored for a range of applications, including synthesizing artificial proteins by genetic code expansion. We review some of these developments, including the ongoing rewiring of the MultiBac system for mammalian applications, notably CRISPR/Cas9-mediated gene editing.

Multiprotein complexes are vital cornerstones of most, if not all, biological processes in cells. To understand the essential mechanisms of these molecular machines, a detailed understanding of their architecture and interactions is required. Several complexes, such as ribosomes, can be purified from native source material in sufficient quality and quantity for structural analysis. Other complexes have low endogenous abundance, impeding their extraction. Heterogeneity can further complicate protein complex purification and structure elucidation. In higher eukaryotes in particular, many complexes can contain distinct subunit isoforms in variable combinations in different tissues. Moreover, the subunit composition can be altered according to cell state. For these complexes, recombinant production and purification by using heterologous host cell systems is typically required.

We encountered this challenge over a decade ago, when working with Tim Richmond in his laboratory at ETH Zürich on multiprotein complexes that control transcription initiation in higher eukaryotes, including humans (reviewed in [1]). Transcription is central to all biological function, and more than a hundred proteins contribute to its regulation. In humans, many of these are organized in multiprotein complexes with ten or more subunits. Post-translational modifications that may require authentic recapitulation in heterologous expression experiments are likewise prevalent. Moreover, subunits can be very large, with individual molecular weights exceeding 200 kDa. After substantial trial and error, we realized that heterologous expression in *Escherichia coli*, which dominated recombinant expression then and still is a preferred choice for many today, was unsuitable for the task at hand. We then turned our attention to eukaryotic methods, specifically an expression vector system relying on a recombinant baculovirus, to infect insect cell cultures for producing the complexes in which we had interest.

## MultiBac origins

Baculovirus was originally trialed as a pesticide, to control crop damage caused by *Spodoptera frugiperda* (the fall armyworm), with limited success initially, although more positive results have recently been reported [2–5]. Subsequently, pioneering work, in particular by Max Summers and colleagues, revealed that baculovirus is useful for very high-level expression of heterologous genes in insect cell laboratory culture (reviewed in [5, 6]). Among the many advantageous features of the baculovirus/insect cell system, the large DNA cargo capacity was particularly notable. Baculovirus, in contrast to other viruses that have a crystalline shell, has a flexible envelope that can grow with increasing size of the genome packaged within the baculovirion without adversely affecting baculovirus function. This particular feature, and the—at least conceptually—relative ease of manipulating baculovirus in the laboratory by non-expert users, was exciting and led us on to the development of MultiBac: a baculovirus/insect cell system specifically engineered for expressing functional multiprotein complexes in the quality and quantity required for high-resolution structural and mechanistic studies [7–11]. MultiBac went on to be broadly adopted and used for research in both industry and academic institutions [12].

The MultiBac system, and its application to protein complex production, has been described in considerable detail previously [8–11, 13]. In summary, MultiBac consists of an engineered baculoviral genome, which is based on the *A. californica* multiple nuclear polyhedrosis virus (AcMNPV). This genome was previously adapted for propagation and manipulation in bacterial cells in the form of a bacterial artificial chromosome (BAC) [14, 15]. Starting from this BAC, we progressively removed functionalities that we identified experimentally to be detrimental to protein complex production. For instance, proteolytic and apoptotic viral factors were eliminated [10]. Baculovirus infection of insect cells is a lytic process as the virus remodels the host cell machinery for producing baculovirions at high levels, resulting ultimately in cell death and lysis. In fact, the highest level of production of the recombinant protein of interest coincides with the onset of widespread cell lysis, which can compromise the protein produced. The introduced genome alterations resulted in a virus that exhibited delayed lysis of the insect cells, allowing the production of recombinant protein complexes at very high levels while the cells seemingly remained intact [11].

**Fig. 1.**
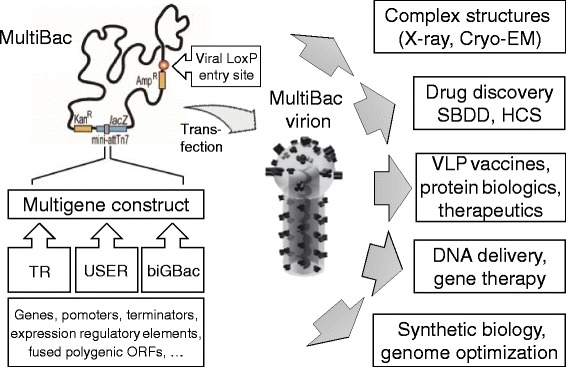
The MultiBac baculovirus expression vector system. MultiBac consists of a baculoviral genome that we engineered for optimal multigene delivery and protein complex expression (*left*). The MultiBac genome is propagated as a bacterial artificial chromosome (BAC) in *Escherichia coli* cells that supply the Tn7 transposition function from a co-existing helper plasmid. Several methods have been successfully applied to assemble expression cassettes containing genes of interest and gene regulatory elements. We introduced tandem recombineering (*TR*), a method that relies on iterative cycles of sequence and ligation independent cloning (SLIC) coupled to Cre-mediated fusion of plasmid DNA precursor elements [9, 19]. Other alternatives include the uracil-specific excision reagent (*USER*) and biGBac methods [20, 21], in addition to conventional restriction/ligation-based cloning techniques. We engineered a second entry option into the viral backbone, which can accept additional functionalities by site specific recombination into the viral *LoxP* site. Composite MultiBac baculoviral DNA containing all DNA elements of interest is extracted. Transfection of insect cell cultures in small scale yields live MultiBac virions. These can be used for a wide range of applications (*right*). The viral LoxP site is shown as a *circle* filled in *red. ORF* open reading frame, *SBDD* structure-based drug design, *HCS* high-content screening, *VLP* virus-like particle, *Cryo-EM* structure determination by electron cryo-microscopy, *X-ray* X-ray crystallography, *Kan* kanamycin resistance marker, *Amp* ampicillin, *LacZ* LacZα gene enabling blue/white selection, *mini-attTn7* attachment site for Tn7 transposition. The schematic drawing of the baculovirion was kindly provided by Kari Airenne

Heterologous genes of interest are inserted into the MultiBac baculoviral genome by means of transfer plasmids, which are transformed into so-called DH10MultiBac cells harboring the BAC (Fig. 1). We prepared an array of small synthetic plasmid DNA precursor modules to generate multigene transfer plasmids that were easily inserted into the MultiBac genome by using tandem recombineering (TR), which can optionally be done in high-throughput [13, 16–19]. TR is a method we conceived to facilitate DNA assembly by iteratively exploiting sequence and ligation independent cloning (SLIC) to insert genes of interest into individual plasmid DNA precursors. These are then concatenated by Cre-*LoxP*-mediated plasmid fusion and then inserted into the MultiBac baculoviral genome by transposition, catalyzed by the Tn7 transposase. The transposase is provided in the DH10MultiBac cells from a separate helper plasmid co-existing with the BAC. Although we mostly use SLIC to manipulate our heterologous genes of interest, our plasmid modules also contain multiple cloning sites for conventional restriction/ligation cloning. Moreover, others have provided valuable alternatives (such as uracil-specific excision reagent (USER) cloning and biGBac) to assemble large transfer plasmids that contain many genes for integration into the MultiBac baculoviral genome [20, 21] (Fig. 1). The USER approach, for instance, is a ligation-independent cloning method utilizing the incorporation of uracil in the primers to delimit sticky overhangs generated by exonuclease treatment; USER facilitates assembly of multigene expression constructs for producing the APC/C multiprotein complex [20]. The biGBac method provides a selection of thoroughly validated primer DNAs for multigene expression cassette assembly relying on the Gibson method, and facilitated reconstitution of the kinetochore complex [21]. Note that TR, USER and biGBac (and other approaches such as conventional restriction digestion/ligation) are not mutually exclusive methods to assemble DNA into multi-expression cassettes for insertion into MultiBac baculoviral genomes but can be used in combination according to the requirements of the project.

## Accelerating multiprotein complex structural biology

**Fig. 2.**
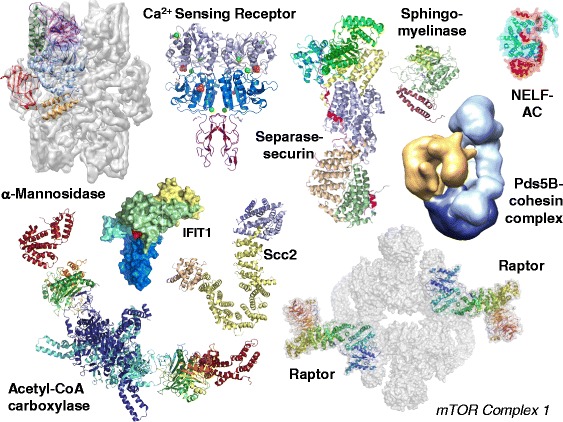
Multiprotein complex structural biology. A selection of recent near-atomic structures of MultiBac-produced specimens, determined by X-ray crystallography or electron cryo-microscopy [22–31]. In the case of mTOR complex 1, heterologous Raptor was produced by using MultiBac and crystallized, and the crystal coordinates fitted into a cryo-EM map of endogenously purified holo-complex in a hybrid approach [31]. Accession numbers from *top left* to *bottom right* are EMDB 8166 (α-mannosidase), PDB 5K5S (Ca^2+^ sensing receptor), PDB 5U1T (separase-securin complex), PDB 5FIB (sphingomyelinase), PDB 5L3X (NELF-AC), PDB 5I6I (acetyl-CoA carboxylase), PDB 5UDL (IFIT1), PDB 5ME3 (Scc2), EMDB 4029 (Pds5B-cohesin complex) and PDB 5FLC (mTOR complex 1)

**Fig. 3.**
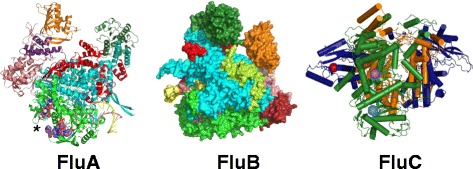
Flu polymerase structures. Crystal structures of MultiBac-produced polymerases of the three major influenza subtypes, fluA, fluB and fluC, have been determined [32–35]. For fluA and fluB, a polyprotein approach was used to balance stoichiometry. Subunits are expressed as a single open reading frame (ORF) encoding a large polyprotein, which is then processed by highly specific protease included in the ORF [18, 37]. By contrast, fluC was crystalized using conventional co-expression of the subunits. FluA and fluB polymerase in the crystals are bound to cognate RNA; the fluC structure represents the apo form of the complex. More recently, the molecular interactions between fluA polymerase and a short C-terminal domain (CTD) peptide from RNA polymerase II (Pol II) were elucidated [34] (*left*). The *asterisk* denotes the position of Pol II CTD peptides bound. PDB accession numbers are: fluA polymerase, PDB 5M3H; fluB polymerase, PDB 4WSA; fluC polymerase, PDB 5D9A

MultiBac was first adopted by the structural biology community and has enabled structure elucidation of many multiprotein complexes, illuminating their cellular functions. Here, we show a selection of recent structures obtained with MultiBac-produced specimens [22–31] (Fig. 2). Noteworthy in this context are the high-resolution structures of influenza polymerase—the long-elusive complex that the influenza virus uses to replicate and transcribe its genome [32–35]. Influenza polymerase consists of three subunits, PA, PB1 and PB2, with many functions, including cap-snatching and endonuclease activities. Since its discovery decades ago, influenza polymerase has been studied intensively, but atomic resolution structures of this coveted drug target have remained elusive owing to lack of high quality samples. Influenza polymerase production by using the MultiBac system allowed the polymerase to be crystallized. The crystal structures of polymerases from three major influenza types, A, B and C, were determined, revealing functional aspects of these protein machines in unprecedented detail (Fig. 3). Interestingly, efficient production of influenza A and B polymerases pre-necessitated the implementation of a polyprotein approach mimicking the strategy certain viruses, such as coronavirus, exploit to realize their proteome. In our polyproteins, the genes encoding the subunits of the complex studied are combined in a single large open reading frame (ORF). This ORF is integrated into the MultiBac genome, giving rise to a large protein that is subsequently processed by a highly specific protease (in our case TEV NIa) that is likewise encoded by the ORF. The protease cleaves specific recognition sites present between the subunits, thus releasing them in a precise stoichiometric ratio, resulting in the functional complex [8, 36, 37].

## MultiBac evolution: synthetic viral nanosystems

**Fig. 4.**
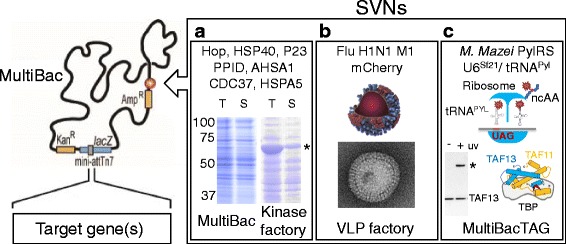
Synthetic viral nanosystems. Synthetic viral nanosystems (*SVNs*) are created by customizing MultiBac baculoviral genomes through integration of additional functionalities into the viral *LoxP* site. Selected examples are depicted in the *box* on the *right* (panels a–c). **a** TheMultiBac-based “kinase factory” contains seven chaperones that assist in the folding of kinase enzymes, thus enhancing the stability and yield of functional protein. Coomassie-stained SDS-PAGE sections show test expression of KRS1, with the MultiBac virus (*left lanes*) and the kinase factory (*right lanes*), respectively. *T* total cell extract, *S* soluble protein. The *asterisk* denotes KRS1, which was expressed as a glutathion-S-transferase (*GST*) fusion protein. Molecular weight marker band sizes (in kDa) are indicated by numbers. The kinase is barely visible in the MultiBac expressions. By contrast, high-level expression of kinase is observed with the kinase factory. **b** “Virus-like particle (*VLP*) factory” was constructed by integrating the gene encoding influenza H1N1 M1 capsid protein into the viral *LoxP* site, together with the florescent protein mCherry-encoding gene to track virus performance. The VLP factory efficiently produces influenza virus-like particles decorated with envelope proteins co-expressed from the viral Tn7 site. VLP is shown in a schematic drawing (courtesy of D. Jordan), with the capsid formed by M1 protein colored in *brown*. The VLP is devoid of genetic content but displays hemagglutinin (*blue spikes*) and neuraminidase (*purple spikes*) on its surface in a live-virus-like fashion, embedded in a host-cell derived lipid bilayer envelope. An EM image of influenza VLP produced by using VLP factory is shown beneath the schematic. **c** The MultiBacTAG SVN. Orthogonal pyrolysyl tRNA (*tRNA* ^*Pyl*^) and cognate synthetase (*PylRS*) from *Methanosarcina mazei* inserted in the viral *LoxP* site enable integration of artificial (non-canonical) amino acids (ncAA) by UAG stop-codon suppression. The growing polypeptide chain contains the artificial amino acid (ncAA, *red star*) at the position determined by the UAG codon. MultiBacTAG was used to integrate a UV-activatable amino acid into a protein complex formed by human transcription factors TAF11 and TAF13, revealing specific crosslinks in the presence of TBP (drawn schematically in *gray*). A section from a western blot stained with anti-TAF13 antibody shows the cross-link upon UV radiation. Reprinted by permission from Macmillan Publishers Ltd: Nature Methods 13:997-1000, copyright 2016 [58]

When MultiBac was initially conceptualized, the DNA sequence *LoxP* was engineered into the viral backbone. This provided the means to integrate further genes of interest by Cre-*LoxP* fusion into this second locus, distal to the Tn7 attachment site. Our incentive was to provide the option for distributing the genes encoding a multiprotein complex, in case it became inconvenient to integrate many expression cassettes into a single site on the viral backbone. This turned out to be less of a concern than we had anticipated, and therefore this viral *LoxP* site was initially used only to integrate a fluorescent marker, yellow fluorescent protein (YFP). This allowed real-time virus performance to be tracked and the optimal timepoint for harvesting the cell cultures to be determined [13]. In addition, the viral *LoxP* site turned out to be useful for introducing specific functionalities, giving rise to customized genomes with tailored functions (Fig. 4), which we termed synthetic viral nanosystems (SVNs).

With the Grabherr group in Vienna, we created SweetBac, a SVN designed to tailor the glycosylation pattern of secreted proteins expressed in insect cells [38–41]. By exploiting the viral *LoxP* site, mammalian glycosylases were integrated into the BAC, which converted the insect-cell-specific high-mannose type glycosylation into the complex sugars associated with human proteins. Conversely, by introducing the deglycosylases PNGase or EndoH, respectively, we created SVNs that entirely removed sugars from the recombinant proteins. This is often preferred for structure elucidation by X-ray crystallography, in which a highly homogenous sample is required, ideally lacking variable extensions, such as sugars, which could impede crystallization. A further post-translational modification that can lead to heterogeneity is phosphorylation, which may or may not be authentic in insect cell expression. In our study of Isw, a chromatin remodeling enzyme complex, we found that the SVN that expresses λ-phosphatase from the viral *LoxP* site could remove the phosphate groups quantitatively from the recombinant complex, resulting in a highly homogenous sample that could be dissected mechanistically [42, 43].

Following the same logic, further SVNs were developed for improved manufacturing of selected classes of protein biologics of pharmacological interest. Kinases are cornerstones of signal transduction in cells, and many disease states, notably cancer, are caused by malfunction of kinases and their interactions. Small-molecule drugs acting as kinase inhibitors are prolifically used in cancer chemotherapy, and the discovery of new and better kinase inhibitors is a major driving force in oncology research and development. A vital prerequisite to sustain these efforts is the supply of high-quality recombinant kinases for screening and structure-based design approaches. Kinases are often inherently fragile proteins that require helper molecules, such as chaperones, for proper folding during expression. To enhance recombinant production of properly folded and soluble kinases, we designed SVNs that contain up to seven chaperones in the viral *LoxP* site (Fig. 4). To balance the quantities of individual chaperones produced, we again applied our polyprotein strategy. The resulting “kinase factory” produced previously unobtainable kinases at high levels, in soluble and functional form.

Another class of protein biologics of acute interest are virus-like particles (VLPs), which mimic live viruses but are safe and non-infectious as they lack genetic content. VLPs therefore represent promising candidates for vaccination to combat infectious diseases [44–46]. The influenza M1 capsid is a powerful driver of VLP formation in insect cells. We therefore integrated a gene that expressed M1 from influenza strain H1N1 into the viral *LoxP* site, together with a gene encoding the fluorescent protein mCherry, which we used to monitor virus performance (Fig. 4). We used this SVN for high-throughput production and validation of an array of influenza VLPs with a large number of immune-modulating mutations in their hemagglutinin (HA) gene [47].

An unusual application of MultiBac technology involved the generation of recombinant adenovirus associated vector (rAAV) for gene therapy [48–51]. Here, MultiBac was used to provide all components (REP78, REP72, AAV virion coat proteins and the transgenes) required to co-produce gene-therapy-competent rAAVs in Sf9 insect cell cultures. The components spontaneously assemble to form intact rAAVs. The system was used to produce rAAVs encoding leptin, for gene therapy of obesity in laboratory rodents. Although the gene therapy successfully resulted in weight-loss, it was nonetheless noted in the report that best results were achieved when the gene therapy regimen was accompanied with exercise using a tread-mill [48], somewhat dampening optimism that exercise-free weight loss may be achievable. Originally, this approach involved co-transfection of three individual MultiBac viruses to obtain intact rAAVs, constraining logistics. More recent refinement of the system, including integrating the encoding genes directly into the genome of the host Sf9 cells, resulting in a streamlined manufacturing process representing a viable alternative for rAAV production [48–51].

## MultiBacTAG: extending the scope of genetic code expansion

Genetic code expansion (GCE) incorporates artificial amino acids into polypeptide chains to create synthetic proteins with novel functions; it has many applications, ranging from discovery science to molecular medicine [52–54]. In GCE, an orthogonal tRNA/cognate synthetase pair is used to suppress a rare stop codon introduced at a specific site in a gene of interest. The tRNA acts as a stop codon suppressor, effectively repurposing the rare stop codon of choice into a functional sense codon during protein synthesis, which leads to the site-specific incorporation of an artificial amino acid supplemented in the culture medium. A wide range of functionalities from protein labeling to photo-control can be introduced by using GCE in biotechnology and biomedical research [55–57]. Until recently, this method has been mostly confined to small individual proteins, expressed in *E. coli* or mammalian cells, representing a limited repertoire of cellular activity. Together with the Lemke and Braese groups, we extended the scope of this method by combining GCE with the MultiBac system. This gave rise to MultiBacTAG (Fig. 4), a protein engineering platform that enables GCE in complex multiprotein machines expressed in insect cells [58].

To customize the MultiBac baculovirus for GCE, the pyrolysyl tRNA/tRNA synthetase (PylRS/tRNA^Pyl^) pair from *Methanosarcina mazei* was chosen. The gene encoding the synthetase and a cassette for producing tRNA^Pyl^ was inserted into the viral *LoxP* site. The design of the tRNA production cassette proved to be surprisingly complicated. The available insect RNA polymerase III-dependent promoters for RNA production (*Bombyx mori* U6 and *Drosophila melanogaster* U6) did not function in the *S. frugiperda* Sf21 cells that we typically use for MultiBac-based protein production. Overcoming this bottleneck required sequencing the Sf21 genome to identify a Sf21 U6 promoter which supported efficiently the production of *M. mazei* tRNA^Pyl^_,_ leading to effective rare stop codon suppression and incorporation of artificial amino acids derived from pyrolysine into the proteins expressed with the MultiBacTAG SVN.

Current MultiBacTAG applications include artificial amino acid cross-linking to map interactions in protein complexes, fluorescence labeling of specific targets to measure structure and dynamics in proteins and glycol-engineering proteins compatible with human tissue studies. We anticipate that the platform will also be adopted for custom-design proteins for therapeutic biotechnology and pharmaceutical applications. As an example, we used MultiBacTAG to engineer trastuzumab (also known as Herceptin), an antibody that associates with cancer cells, to recognize breast cancer cells in human tissue [58, 59].

## MultiBac-based genome engineering by CRISPR/Cas9

Multigene delivery and subsequent cellular expression is a key technology for a wide range of applications in biology, not only in structural research but also cellular reprogramming and functional pharmaceutical screening. The construction of multigene circuits in mammalian cells is a core concept in synthetic biology and requires efficient delivery of complex heterologous DNA. For certain cell types, including the widely used HEK293 and HeLa cells, this can be achieved by plasmid-based transfection. However, many cell lines, including primary cells, are recalcitrant to plasmid transfection and therefore require a different approach. Baculovirus in its original form is highly selective for infecting insect cells, which is among the reasons why baculovirus/insect cell expression can be carried out at standard laboratory biological safety levels. At very high concentrations, however, baculovirus can also penetrate mammalian cells. This process is called transduction rather than infection as the baculovirus, in contrast to mammalian viral pathogens, will not replicate in mammalian cells. If the baculoviral genome that has been transduced into the mammalian host cell contains an expression cassette with a mammalian cell active promoter, expression of the transgene occurs [60, 61]. This “BacMam” gene delivery approach is attractive owing to the very large DNA cargo capacity of baculovirus. In principle, a single baculovirus can deliver many genes of interest at the same time, a result that is difficult to achieve by plasmid-based co-transfection.

**Fig. 5.**
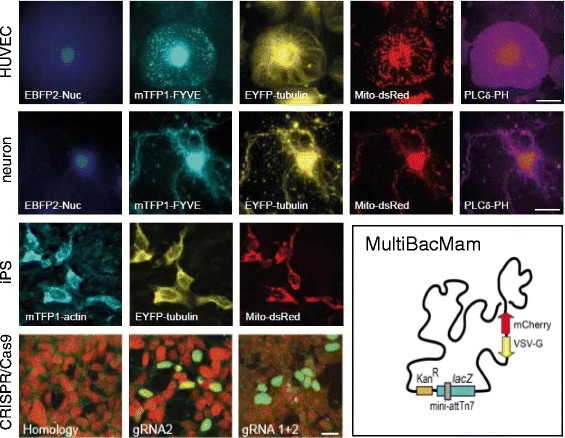
Mammalian multigene delivery and gene editing. The tropism of the MultiBac baculovirus was expanded by pseudotyping. A gene encoding for vesicular stomatitis virus glycoprotein (VSV-G) was integrated into the viral *LoxP* site, accompanied by a mCherry gene for tracking virus performance, resulting in the MultiBacMam virus (*bottom right*). MultiBacMam efficiently transduces mammalian cell types known to be recalcitrant to plasmid-based transfection, such as human umbilical vein endothelial cells (*HUVECs*), neurons and induced pluripotent stem (*iPS*) cells, with multigene DNA cargos encoding multiple fluorescently labeled proteins (*top three rows*). Transduction with MultiBacMam virus comprising the elements required for CRISPR/Cas9-mediated gene editing and a GFP-encoding gene as model DNA cargo resulted in successful integration into the HMG locus (*bottom row*) [63]

We customized the MultiBac baculovirus by integrating the gene encoding vesicular stomatitis virus G protein (VSV-G), a protein which was shown to substantially increase the transduction efficacy [62]. The resulting MultiBacMam baculovirion displays VSV-G glycoproteins on the virion surface, and was well suited for transducing a wide range of established mammalian cells with unprecedented efficacy [63]. Primary cells are a central focus of current biological research efforts, and multigene delivery in primary cells is highly desirable; however, suitable tools have been markedly lacking. We demonstrated that MultiBacMam potently transduced primary cells (Fig. 5). Moreover, MultiBacMam could be used to reprogram fibroblasts into neurons, with virtually identical efficiency as a lentiviral approach. Of note, MultiBacMam transduction is transient in nature and does not permanently alter the genome, in contrast to the genomic integration of lentivirus.

The very large DNA cargo capacity of the baculovirion, and its aptitude to transduce mammalian cells, raises the attractive possibility to use this tool, in conjunction with powerful gene editing technologies, to rectify genetic aberrations, possibly in whole organisms. The gene encoding Cas9 and the modalities to produce guide RNAs and a gene encoding eGFP as a model DNA cargo were integrated into the MultiBacMam system. We showed that this MultiBacMam genome engineering tool could be used for CRISPR/Cas9-mediated insertion of eGFP into the HMG locus of a range of cell types, including neurons [63]. Thus, MultiBacMam can now be developed into a bona fide genome-engineering tool, characterized by an unsurpassed DNA cargo capacity that is orders of magnitude larger than the commonly used gene therapy viral vectors (lentivirus, AAV and adenovirus) that currently dominate the field.

## MultiBac Redux: SynBac

The original AcMNPV genome from which the MultiBac is derived contains all the information required for the virus to sustain its life cycle in nature, starting from uptake by the host (the fall armyworm), the infection of midgut cells, followed by massive amplification and release of occluded viral particles when the worm lyses. Many of these functions are dispensable, or even detrimental, in laboratory cell culture. The advent of powerful DNA synthesis and assembly technologies has now made it feasible to “rewire” entire genomes. Genome improvement by eliminating undesired functionalities is a major ambition in current synthetic biology. The baculoviral genome is around 140 kb, which is the size of an average BAC, and within the size that is capable of complete de novo synthesis, as recently shown [64].

A disadvantage of currently available recombinant baculoviral systems, including MultiBac, is genome instability during virus amplification. The baculovirus progressively eliminates DNA from its genome when serially passaged over several generations, often most severely affecting the heterologous DNA to be expressed [65–67]. In extreme cases, only a few full-size baculoviral genomes are present after several rounds of amplification in cell culture, and small circular DNAs containing tiny fractions of the baculoviral genome dominate. These survive by scavenging on the packaging proteins produced from the full-size specimens, overtaking the viral population. For academic research requiring typically only a few milligrams of protein sample, this can be controlled in a relatively straightforward manner by avoiding virus overamplification [9, 10, 68]. However, this is much more difficult and often impossible to achieve at scales relevant for pharmaceutical manufacturing. Here, large volumes of virus and therefore many cycles of amplification are required to infect fermenter-scale production runs. An improved baculoviral genome resolving this handicap would thus be a major advance.

**Fig. 6.**
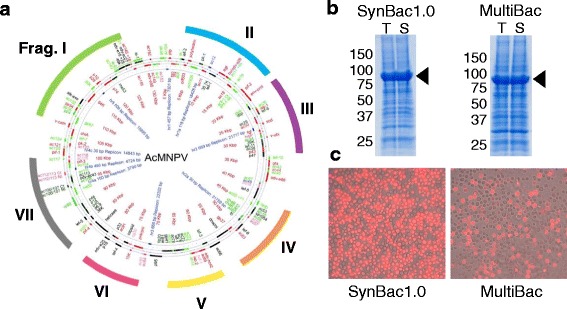
Minimizing and optimizing the baculoviral genome to create SynBac. **a** The DNA elements in the wild-type AcMNPV baculoviral genome were classified according to perceived importance for cell culture applications, derived from exhaustive comparative genome analysis and data mining [69], and distributed into fragments (I to VII) for rewiring and condensing, with the aim to eliminate surplus DNA and improve virus performance in the laboratory and in manufacturing. **b** Experiments with SynBac1.0, a MultiBac-derivative comprising a condensed Fragment I, showed powerful expression of a test protein (marked by *arrowhead*), indistinguishable from MultiBac based on Coomassie-stained SDS-PAGE. Numbers indicate molecular weight marker sizes (in kDa). *T* total cell extract, *S* soluble protein. **c** Serial passaging of SynBac1.0 expressing a large polygenic test open reading frame (ORF) containing a red fluorescent protein marker (dsRED) evidenced a remarkable increase of SynBac1.0 genome stability compared with MultiBac. More than 70% of infected cells still produce dsRED in fluorescent micrographs, indicating the presence of intact genomes. By contrast, identically overamplified MultiBac virus frequently lost dsRED, resulting in a much lower proportion (<20%) of infected cells producing dsRED

We carried out a comparative study of all available baculoviral genomes in sequence data bases and combined this with data mining to delineate a putative minimal baculoviral genome, SynBac, that would be capable of sustaining its propagation and high-level heterologous protein production in cell culture [69]. Based on our data, the baculoviral genome was divided into segments to be individually streamlined through the elimination of DNA elements that we identified as dispensable for our purposes (Fig. 6). The maximally condensed synthetic DNA sections can then be subsequently grafted back into the MultiBac baculoviral genome to replace the wild-type sequence, and tested for functionality. Synthetic sections that pass rigorous validation are then iteratively combined until the minimal, fully rewired SynBac baculoviral genome is accomplished. We showed that 30 kb of the MultiBac baculoviral genome could be altered in this way. The first-generation virus generated (SynBac1.0) was not only fully functional in terms of high-level protein production, but already showed a major improvement in genome stability during serial passaging of the virus (Fig. 6). This validated the approach and addressed one of the major current shortcomings of baculoviral expression, genome instability.

## Conclusion and outlook

The MultiBac system was shown in its first configuration in 2004 [11], which had been developed to address the challenge of efficient multiprotein complex production for structural biology applications. Subsequently, we received requests from laboratories in both academia and industry exemplifying the need for such a tool-kit, a trend that has not since subsided. The numerous important structures elucidated using MultiBac-produced samples evidenced how useful the system is. We, and others, invested considerable effort over the years to streamline MultiBac, rendering it more user-friendly and accessible, with improvements such as alternative methods for inserting genes of interest.

More recently, the scope of the system has been expanded by customizing the genome for specific applications, as well as altering the tropism of the virus to enable efficient multigene transfer in a wide range of mammalian cell types and organisms. In our view, genomic intervention mediated by nucleases (e.g. Cas9, TALENs and Zinc-fingers), is a particularly promising application. Combining gene editing with the very large (>100 kb) heterologous DNA cargo capacity of baculovirus could be instrumental for future gene therapy applications. We anticipate that next-generation genomic intervention strategies will critically depend on providing complex multicomponent functionalities, including targeting activities and host immune-system modulators in a single multifunctional DNA delivery tool. SynBac, the synthetic baculovirus, could be a tool of choice, providing currently unmatched DNA cargo capacity in an optimized genome.
